# Supplementary figures and images for: Redundant and nonredundant organismal functions of EPS15 and EPS15L1
Source: Life Sci Alliance. 2019 Jan 28;2(1):e201800273. doi: 10.26508/lsa.201800273 (PMC6350104; doi:10.26508/lsa.201800273)

# Brain lysates of Eps15 WT or KO mice

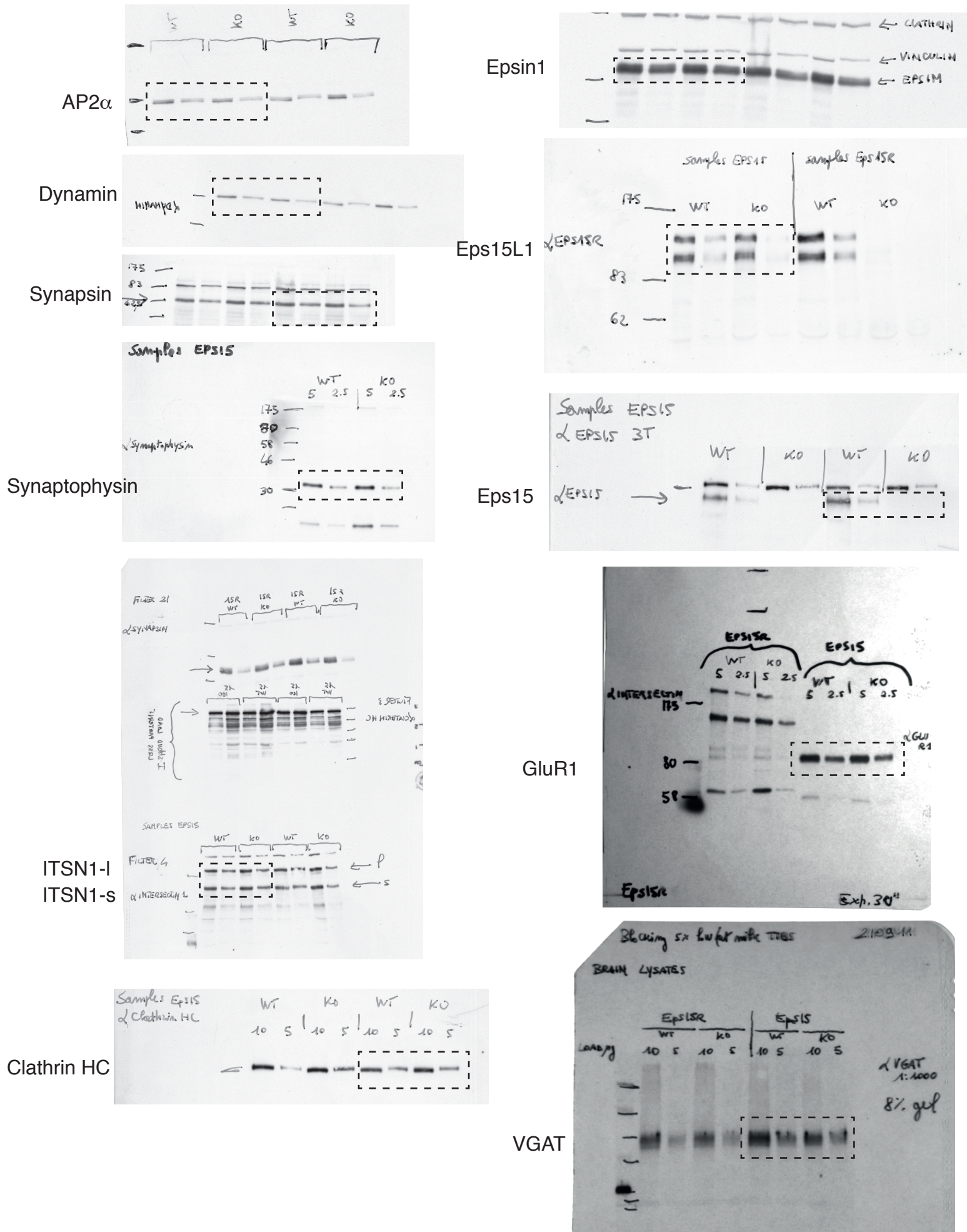

Supplement: Supplementary file 1 [file LSA-2018-00273_SdataF2A.pdf]

Brain lysates of Eps15L1 WT or KO mice

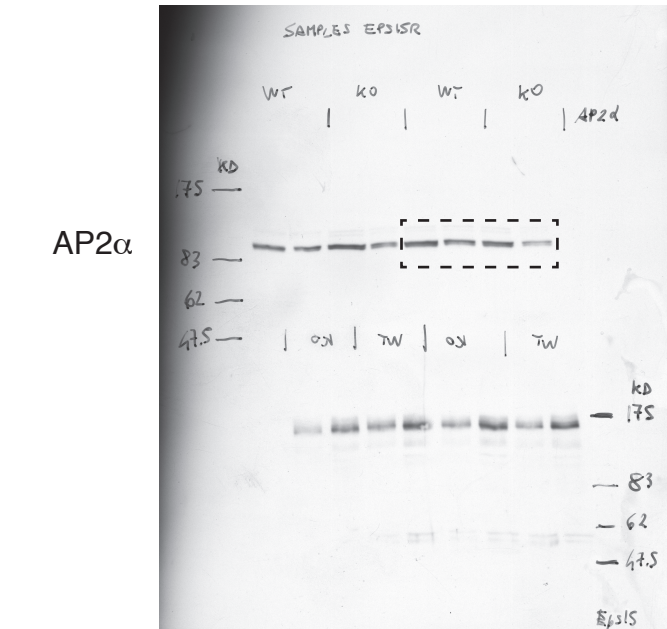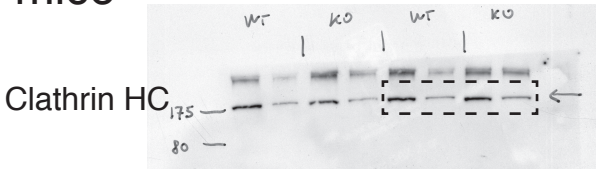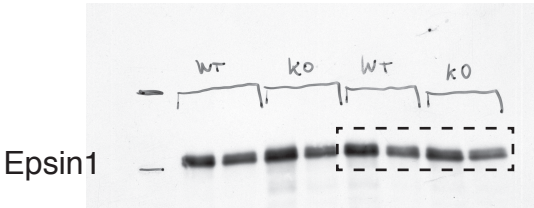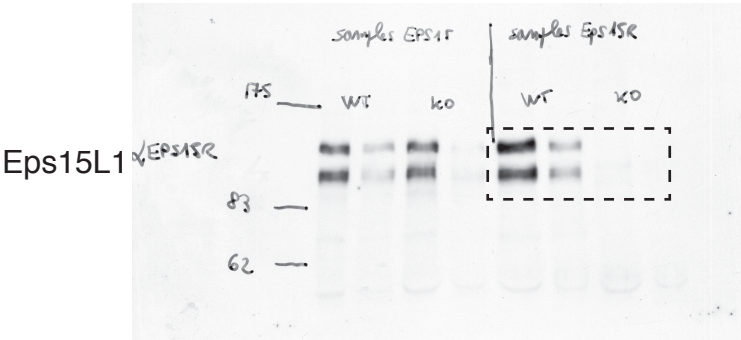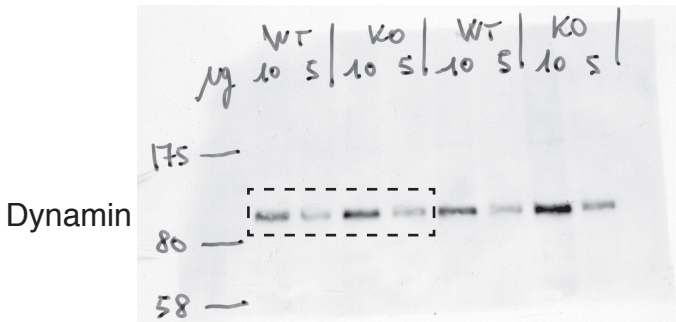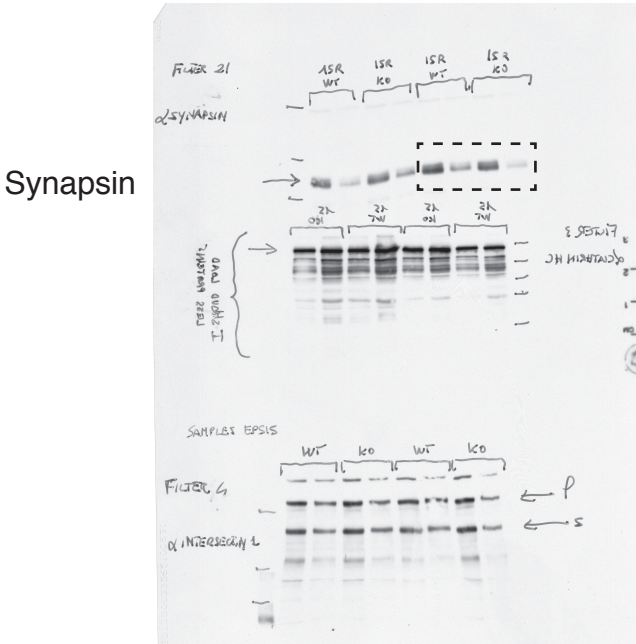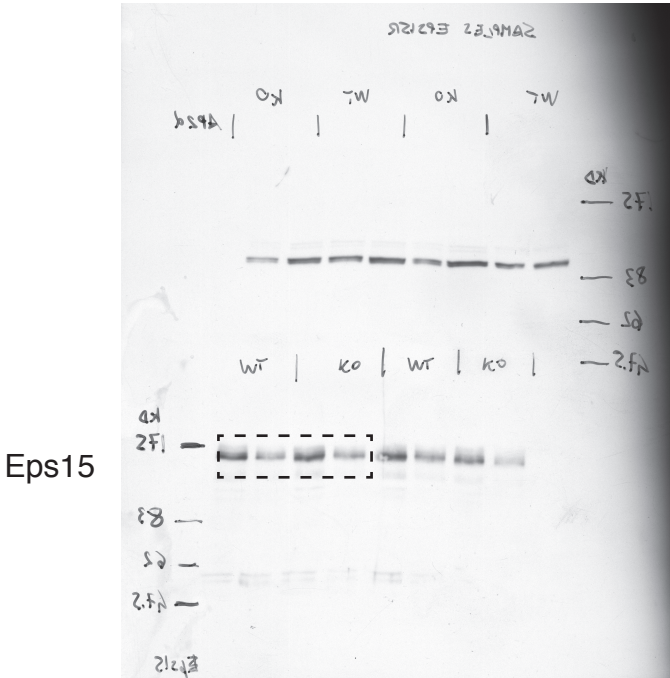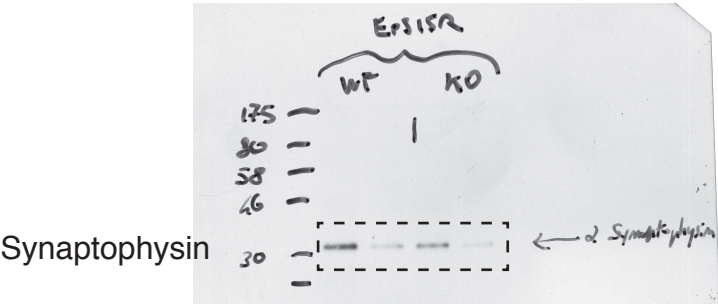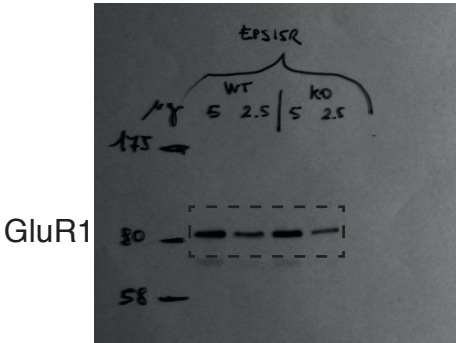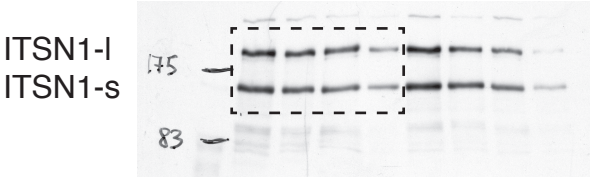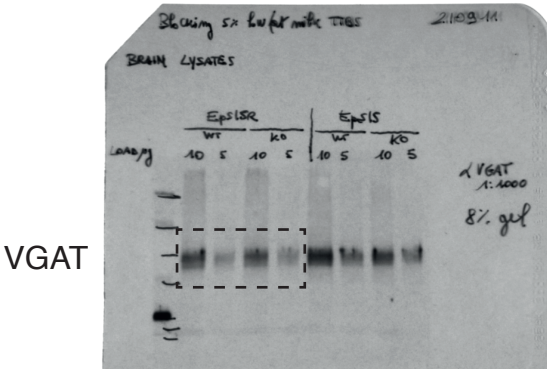

Supplement: Supplementary file 2 [file LSA-2018-00273_SdataF2B.pdf]
